# Developmental features of DNA methylation during activation of the embryonic zebrafish genome

**DOI:** 10.1186/gb-2012-13-7-r65

**Published:** 2012-07-25

**Authors:** Ingrid S Andersen, Andrew H Reiner, Håvard Aanes, Peter Aleström, Philippe Collas

**Affiliations:** 1 Stem Cell Epigenetics Laboratory, Institute of Basic Medical Sciences, Faculty of Medicine, and Norwegian Center for Stem Cell Research, University of Oslo, PO Box 1112 Blindern, 0317 Oslo, Norway; 2 BasAM, Norwegian School of Veterinary Science, PO Box 8146 Dep., 0033 Oslo, Norway

## Abstract

**Background:**

Zygotic genome activation (ZGA) occurs at the mid-blastula transition (MBT) in zebrafish and is a period of extensive chromatin remodeling. Genome-scale gametic demethylation and remethylation occurs after fertilization, during blastula stages, but how ZGA relates to promoter DNA methylation states is unknown. Using methylated DNA immunoprecipitation coupled to high-density microarray hybridization, we characterize genome-wide promoter DNA methylation dynamics before, during and after ZGA onset, in relation to changes in post-translational histone modifications and gene expression.

**Results:**

We show methylation of thousands of promoters before ZGA and additional methylation after ZGA, finding more dynamic methylation -1 to 0 kb upstream of the transcription start site than downstream. The MBT is marked by differential methylation of high and low CpG promoters, and we identify hypomethylated promoters that are mostly CG-rich and remain hypomethylated through the MBT. Hypomethylated regions constitute a platform for H3K4me3, whereas H3K9me3 preferentially associates with methylated regions. H3K27me3 associates with either methylation state depending on its coincidence with H3K4me3 or H3K9me3. Cohorts of genes differentially expressed through the MBT period display distinct promoter methylation patterns related to CG content rather than transcriptional fate. Lastly, although a significant proportion of genes methylated in sperm are unmethylated in embryos, over 90% of genes methylated in embryos are also methylated in sperm.

**Conclusions:**

Our results suggest a pre-patterning of developmental gene expression potential by a combination of DNA hypomethylation and H3K4 trimethylation on CG-rich promoters, and are consistent with a transmission of DNA methylation states from gametes to early embryos.

## Background

DNA methylation is associated with long-term gene silencing and plays an important role in development, × chromosome inactivation and genomic imprinting [1]. In eukaryotes, DNA methylation occurs on cytosines in CpG dinucleotides and is stably inherited through cell division. The mammalian life cycle is marked by two waves of genome-wide DNA demethylation and remethylation [1]. The first occurs during germ cell development, when parental imprints are reset by demethylation and differential methylation of maternal and paternal alleles. The second occurs after fertilization, when maternal and paternal methylation patterns, except for imprinted genes, are erased and re-established during pre-implantation stages. However, at least in the mouse, some non-imprinted genes retain parental promoter methylation and escape post-fertilization reprogramming [2], and over 1,000 methylated CpG islands (CGIs) are also incompletely demethylated even though they are not imprinted [3]. Furthermore, *Xenopus *embryos retain a high methylation level after fertilization [4-6] and show no correlation between promoter methylation and transcriptional repression [6]. These observations suggest a view of maintenance of some sperm methylation patterns after fertilization and illustrate the diversity of methylation options in the embryo.

As in mammals, zebrafish undergoes post-fertilization gametic demethylation and remethylation [7]. Zebrafish embryos develop for ten cell cycles in the absence of transcription until the mid-blastula transition (MBT), at which time zygotic genome activation (ZGA) occurs [8]. Subsequent waves of transcriptional activation and inactivation control early development [9,10]. Shortly after the MBT stage, DNA methylation levels are similar to those of somatic tissues [7,11,12]. The pre-MBT and MBT periods are also characterized by increasing enrichment of the genome in post-translationally modified histones. Genomic occupancy by trimethylated H3K4, H3K9 and H3K27 is initiated prior to ZGA [13] and increases sharply thereafter [13-15]. Histone methylation may therefore cooperate with DNA methylation to shape tissue-specific gene expression [16,17]. It remains unknown, however, how promoter DNA methylation changes throughout the MBT period, how it correlates with gene expression at the time of ZGA, and how it may contribute to pre-patterning developmental gene expression.

Taking advantage of the 3-h transcriptionally quiescent pre-MBT period in zebrafish, we address here whether DNA methylation may pre-pattern early developmental gene expression in the absence of ongoing transcription. We characterize differential changes in promoter DNA methylation before, at the time of, and after ZGA in the context of DNA sequence and H3K4, H3K9 and H3K27 trimethylation, and unravel distinct methylation patterns between cohorts of genes differentially expressed after ZGA. Our data suggest a pre-patterning of developmental gene repression by a combination of promoter DNA methylation and H3 methylation marks.

## Results

### Promoter methylation during the pre-MBT and MBT period primarily targets genes involved in cell signaling

**Figure 1:**
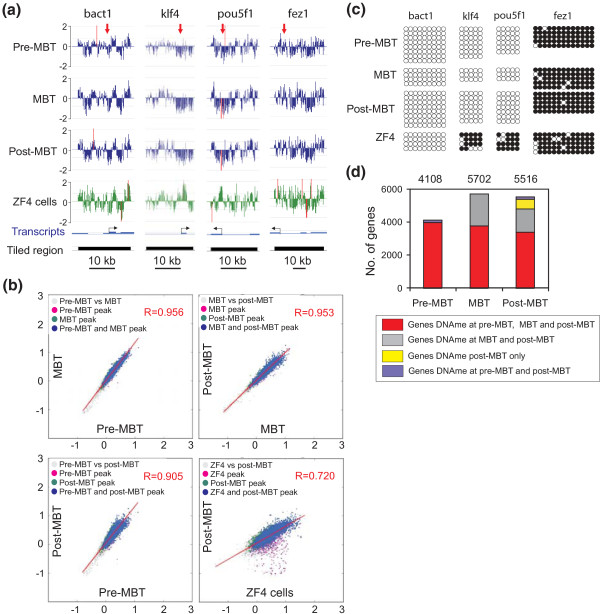
**Promoter DNA methylation states during the transition through the MBT period**. **(a) **MeDIP-chip profiles of DNA methylation in tiled regions spanning a housekeeping gene (*bact1*) and developmentally regulated genes (*klf4*, *pou5f1*, *fez1*) (log2 MeDIP/input ratios), in pre-MBT, MBT, and post-MBT embryos and in the ZF4 fibroblast cell line. Red arrows in the upper track point to regions analyzed by bisulfite sequencing in (b). **(b) **Two-dimensional scatter plots of MaxSixty values for MeDIP log_2_ signal intensities at indicated developmental stages (pairwise) and in ZF4 cells. Average MaxSixty values for both MeDIP replicates are plotted for each stage. Data points are colored to indicate classification according to peak calling algorithm, to show methylated promoters in one only (purple, green) or both (blue) stages. **(c) **Bisulfite sequencing validation of MeDIP-chip data shown in (a); 5' to 3' orientation; filled circles indicate methylated cytosine; empty circles indicate unmethylated cytosine. **(d) **Numbers of methylated genes pre-MBT, MBT and post-MBT. Color reflects genes whose methylation is maintained between stages.

Profiles of promoter DNA methylation were examined in zebrafish embryos before onset of ZGA (2.5 h post-fertilization (hpf); 256-cell stage; 'pre-MBT'), at the time of ZGA (3.3 hpf; high stage; 'MBT') and after ZGA (5.3 hpf; 50% epiboly; 'post-MBT'). Methylation was assessed by methylated DNA immunoprecipitation (MeDIP) and hybridization to microarrays tiling 15 kb upstream and 5 kb downstream of the transcription start site (TSS) of 12,697 zebrafish RefSeq genes [18]. Comparisons of log_2_ MeDIP/input ratios show robust correlation (R > 0.95) between MeDIP replicates and thus high MeDIP-chip reproducibility from early stage embryos (Additional file 1). Hybridization profiles (Figure 1a) and calculated MaxSixty values of methylation intensities for all promoters (Figure 1b) reveal overall similarity between pre-MBT, MBT and post-MBT stages, yet marked differences between embryos and the cultured zebrafish ZF4 fibroblast cell line. MeDIP-chip data were independently validated by bisulfite sequencing, which does not rely on affinity enrichment of methylated DNA (Figure 1c; Additional file 1), and by MeDIP-quantitative PCR (qPCR) for unmethylated and methylated regions (Additional file 1).

Detection of peaks of promoter methylation in a -5 to +1 kb window around the TSS using a Kolmogorov-Smirnov (KS) test with *P *≤ 0.01 identifies a high number of methylated genes at all developmental stages, with 4,108 methylated genes pre-MBT and an increase to 5,702 genes at the MBT (Figure 1d; Additional file 1). Gene ontology (GO) analysis reveals at all stages enrichment of methylated genes in G-protein signaling processes primarily linked to sensory perception (Additional file 2). Several targets for DNA methylation are members of entire gene family clusters (for example, *or*, *taar*), sub-clusters of a given gene family (for example, *opn1 *loci) or individual promoters of the same gene family, located on distinct chromosomes (for example, *chrm*, *fzd*, *gpr *or *adra *family members). This suggests that DNA methylation may have evolved as a mechanism for silencing duplicated promoters, as recently proposed for mouse [2].

### The MBT is marked by differential methylation of high and low CpG promoters

Although the vertebrate genome is generally depleted of CpG dinucleotides, many promoters are protected from this depletion. High CpG promoters (HCPs) are characterized by multiple TSSs and transcriptional activity in multiple tissues, whereas low CpG promoters (LCPs) contain a single TSS and are expressed in a tissue-specific manner [19]. As the average CG content and distribution vary between vertebrate taxa, whether HCPs and LCPs have the same properties in fish as in mammals remains unknown.

To address this issue in a developmental context, we first classified zebrafish promoters into HCPs and LCPs. To this end, we determined the CG content of all promoters on the array within 1 kb upstream of the TSS (Additional file 3) and adapted the Takai and Jones algorithm [20] to the zebrafish observed/expected (o/e) CG ratio and CG content in a 1-kb window upstream of the TSS, to identify HCPs fitting a subsequence with an o/e CG ratio of ≥0.65 and a CG content of >0.30 (Additional file 3). Promoters not matching these criteria were scored as LCPs. Adaptation of the Takai and Jones approach to fish has previously been validated [21], and additional evidence indicates that the divide between HCPs and LCPs is conserved between vertebrate taxa [22]. We identified 7,914 and 4,341 genes in the HCP and LCP class, respectively, making up 65% and 35% of RefSeq genes (Additional file 3). These proportions are similar to those reported for human or mouse promoters [2,23,24].

**Figure 2:**
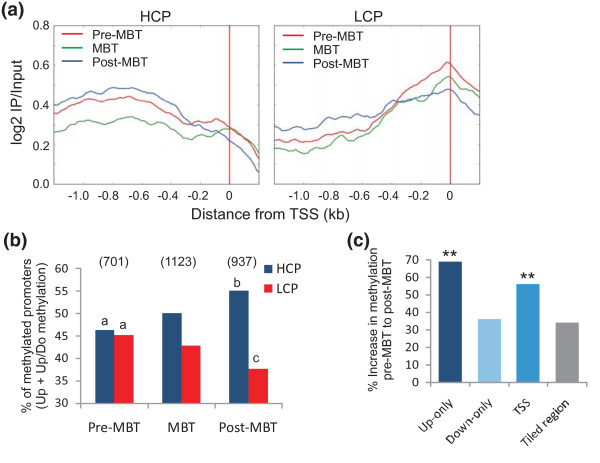
**Characterization of promoter DNA methylation patterns during development through the MBT**. **(a) **Average methylation profiles of HCPs and LCPs at pre-MBT, MBT and post-MBT stages. **(b) **Proportions of methylated HCPs and LCPs at pre-MBT, MBT and post-MBT stages; methylation is defined by detection of a methylation peak in the -1/0 kb window upstream of the TSS irrespective of downstream methylation (upstream (Up) and upstream/downstream (Up/Do) methylation; *y*-axis). ^a,b^*P *= 0.0005; ^a,c^*P *= 0.0027 (Fisher's test). **(c) **Percent increase in the numbers of genes methylated upstream of the TSS (Up-only; -1 to 0 kb), downstream of the TSS (Down-only; 0 to +1 kb), and both upstream and downstream (TSS), from pre-MBT to post-MBT. **P < 0.001 (Fisher) relative to increase in methylation in the whole regions analyzed (-5 to +1 kb).

Average methylation determined in metagene analyses over 1 kb upstream of the TSS among methylated HCPs and LCPs shows that HCPs tend to be methylated upstream of the TSS, whereas LCP methylation peaks over the TSS (Figure 2a). Broadening this analysis to a -2 to +2 kb window on either side of the TSS confirms this interpretation (Additional file 3). Thus, in zebrafish embryos, as in mouse embryos [2], TSS methylation seems to be a hallmark of LCPs. Further, there is no difference in the level of methylation among methylated HCPs and LCPs at any of the stages examined (median methylation level in HCPs versus LCPs, determined by log2 MeDIP/input ratios: pre-MBT, 0.44 versus 0.42; MBT, 0.54 versus 0.46; post-MBT, 0.57 versus 0.47). There is also no difference in the profile of methylation within HCPs and LCPs at the pre-MBT, MBT and post-MBT stages (Figure 2a), indicating overall stability of promoter methylation patterns during this developmental period. However, we note a differential methylation of HCPs and LCPs in the -1 to 0 kb region relative to the TSS, as embryos develop through the MBT (Figure 2b). Whereas HCPs and LCPs are methylated in similar proportions pre-MBT, the MBT and post-MBT stages are marked by a greater increase in the number of methylated HCPs (*P *= 0.0005) than LCPs (*P *= 0.0027) (Figure 2b). Thus, promoters *de novo *methylated during development through the MBT are predominantly HCPs. Therefore, developmentally linked CG-rich promoters may not be as strongly protected from *de novo *methylation in embryonic cells as in more differentiated cells, where they are more resilient to differentiation-induced methylation [24].

As we observed methylation differences upstream and downstream of the TSS of some genes (Figure 1a; Additional file 1), we assessed the developmental changes in methylation -1 to 0 kb upstream of the TSS, 0 to +1 kb downstream, and both -1/0 and 0/+1 kb relative to the TSS (Additional file 4). Average upstream methylation is strongest -1 to -0.5 kb, while downstream methylation peaks at +0.5 to +1 kb. 'Upstream/downstream methylation' reveals enrichment within -0.5 to +0.5 kb of the TSS, a pattern we henceforth refer to as 'TSS methylation'. Globally, genes with a given methylation profile pre-MBT retain this profile at subsequent developmental stages. However, between the pre-MBT and MBT stages, the increase in the proportion of genes with upstream-only methylation or TSS methylation is greater than the increase in the proportion of methylated genes throughout the tiled region examined (*P *< 0.001; Figure 2c). Thus, methylation in the proximal promoter region is particularly prone to *de novo *methylation as embryos develop throughout the MBT period.

### Differential DNA methylation of developmentally regulated gene cohorts

**Figure 3:**
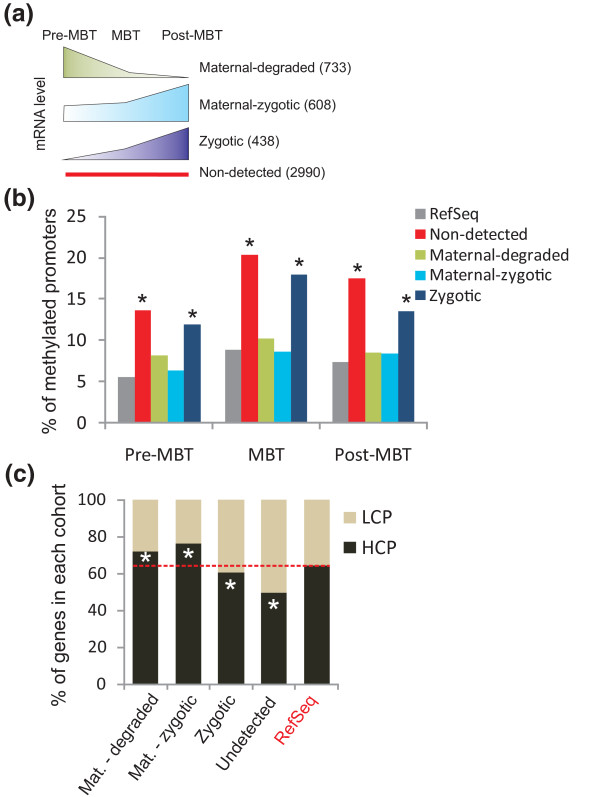
**Relationship between promoter DNA methylation and embryonic gene expression**. **(a) **Schematic representation of the developmental gene expression cohorts previously identified by RNA-seq [10] (numbers of genes encoding the corresponding transcripts). **(b) **Proportions of genes with a methylated promoter (-1/0 kb) in each cohort at the pre-MBT, MBT and post-MBT stages. *P < 10^-4 ^relative to RefSeq genes (Fisher). **(c) **Proportions of LCPs and HCPs in each gene cohort. *P < 10^-4 ^relative to RefSeq genes (Fisher).

We have recently shown that promoter marking by H3K4me3 pre-MBT correlates with a propensity of the corresponding genes to be expressed after ZGA, suggesting that H3K4me3 marking pre-MBT may 'pre-pattern' genes for developmental gene expression [13]. How DNA methylation may contribute to this pre-patterning is, however, undefined. We therefore assessed upstream and downstream methylation at the pre-MBT, MBT and post-MBT stages, among four developmentally regulated gene clusters that we recently defined by RNA-sequencing (RNA-seq) [10] (Figure 3a). These include a 'maternal-degraded' cluster of maternal transcripts detected in unfertilized eggs and declining pre-MBT (733 genes); a 'maternal-zygotic cluster' of maternal transcripts upregulated at the MBT or post-MBT (608 genes); a 'zygotic cluster' of transcripts only detected from the MBT or post-MBT onwards (438 genes); and an 'undetected' cluster of transcripts not detected by RNA-seq at any stage (2,990 genes).

Promoter methylation is most prominent in the undetected cluster, but strikingly also in the zygotic cluster, in which proportions of methylated genes are enriched over that of all methylated RefSeq genes (*P *< 10^-5^; Figure 3b). Thus, as recently reported in *Xenopus *[6], we find no relationship between transcript detection and DNA methylation -1 to 0 kb of the TSS in zebrafish embryos, indicating that promoter DNA methylation is not a key determinant of embryonic gene expression. Maternal genes have the lowest proportion of methylated promoters (Figure 3b), in line with their enrichment in HCPs (*P *< 10^-4^; Figure 3c). Genes of the zygotic and undetected mRNA clusters harbor in contrast a lower proportion of HCPs (Figure 3c). There is, however, no difference in the percentage of HCPs between maternal-degraded and maternal-zygotic gene clusters, indicating that their transcriptional fate is not determined by DNA methylation or CG content.

### Hypomethylated regions are largely conserved through the MBT

**Figure 4:**
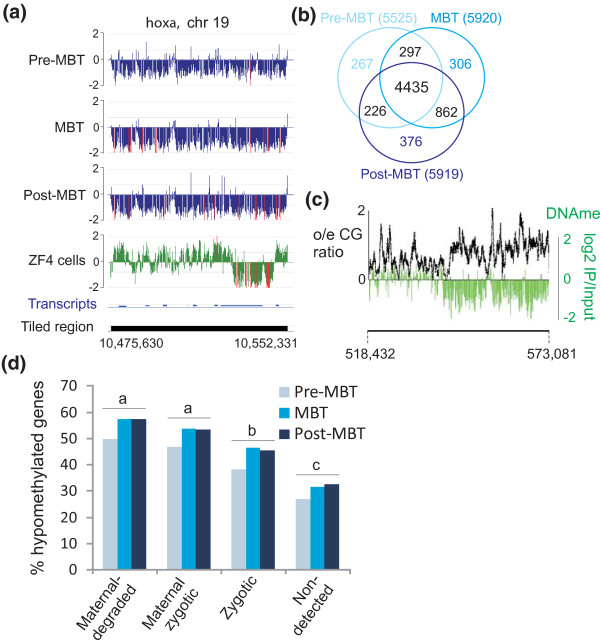
**Maternal genes are preferentially hypomethylated relative to zygotic or non-expressed genes**. **(a) **Hypomethylated domain over the *hoxa *locus in embryos and ZF4 cells. **(b) **Venn diagram analysis of hypomethylated genes at pre-MBT, MBT and post-MBT stages. **(c) **Domains of high o/e CG ratios exhibit hypomethylation, exemplified in a 51-kb region of chromosome 11 in pre-MBT embryos. **(d) **Proportions of hypomethylated genes in developmental gene cohorts. ^a,b/a,c/b,c^*P *< 10^-4 ^within developmental stage (Fisher).

In addition to methylated regions, embryos display domains of hypomethylation, statistically identified as methylation levels below genome average (Figure 4a; see Materials and methods). We identify, pre-MBT, 5,225 hypomethylated promoters (-1 to 0 kb region), which mostly remain hypomethylated at the MBT and post-MBT stages (Figure 4b). Nearly 80% of hypomethylated promoters are HCPs, which represents an enrichment over the proportion of HCPs in RefSeq genes (65%; *P *< 10^-5^). Hypomethylated domains also extend beyond promoters and include larger CG-rich areas (Figure 4c). At each developmental stage, GO term analysis revealed enrichment of hypomethylated genes in transcription regulation, metabolic and developmental functions (Additional file 2). Furthermore, in line with the high CG content of maternal genes, hypomethylated genes are enriched in the maternal-degraded and maternal-zygotic clusters relative to the zygotic and non-expressed clusters, at each stage (*P *< 10^-4^; Figure 4d). Therefore, the hypomethylated state is overall maintained during development through the MBT.

### Hypomethylated promoters constitute a platform for H3K4 trimethylation

Genomic areas enriched in H3K4me3 have been shown in mammalian cells to be devoid of DNA methylation owing to the inability of DNA methyltransferase-like protein DNMT3L to bind to the trimethylated form of H3K4 [25]. However, zebrafish does not seem to harbor a homologue of mammalian *DNMT3L*; thus, whether H3K4me3 regions are exempt from DNA methylation remains an open question. In addition, whether DNA methylated regions are enriched in repressive histone marks is unknown. Thus, we examined DNA methylation profiles in the context of our recent H3K4me3, H3K9me3 and H3K27me3 chromatin immunoprecipitation (ChIP)-chip data using the same arrays as those employed here [13].

**Figure 5:**
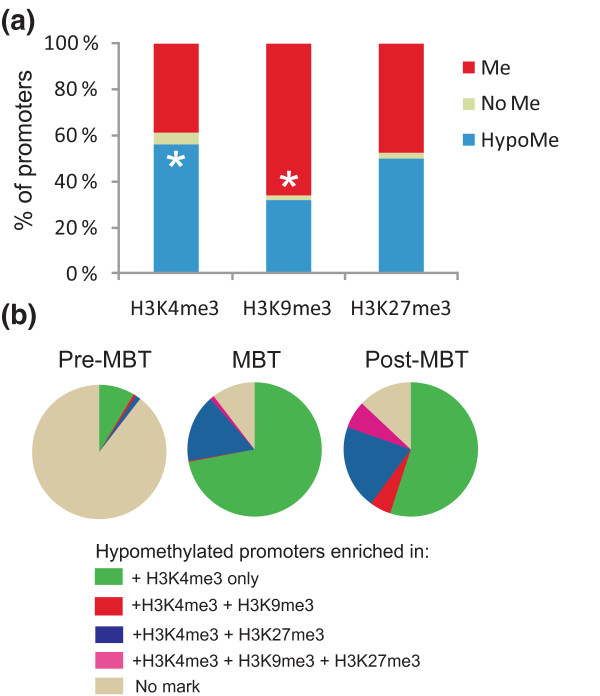
**Enrichment of hypomethylated and methylated genes in, respectively, H3K4me3 and H3K9me3**. **(a) **Post-MBT proportions of H3K4me3, H3K9me3 and H3K27me3 enrichment on methylated promoters, hypomethylated promoters and promoters with genome-average methylation (that is, neither statistically hypomethylated nor methylated; 'No Me'). *P < 10^-4 ^relative to the opposite methylation state (Fisher). Histone methylation data are from our laboratory [13]. **(b) **Proportions of hypomethylated promoters not marked by H3K4me3 (or any other histone modification), or co-enriched in H3K4me3 only or with a repressive mark (H3K9me3, or H3K27me3, or both).

An intersect of histone modifications and DNA methylation data shows that when histone methylation is massively detected on the embryonic genome (post-MBT) [13], H3K4me3 is preferentially localized on hypomethylated promoters (*P *< 10^-5^; Figure 5a). In contrast, H3K9me3 mainly associates with methylated promoters (*P *< 10^-5^), whereas H3K27me3 shows no preference for methylated or hypomethylated promoters (*P *= 0.08). These observations raise the hypothesis that hypomethylated promoters may constitute a platform for H3K4 trimethylation, a condition that has been shown to be sufficient to recruit H3K4me3 in mammalian cells [26]. The majority of these promoters are monovalent for H3K4me3, that is, without any repressive H3K9me3 or H3K27me3, at least up to the MBT stage (Figure 5b). After the MBT, a stage where epigenetic complexity develops through massive enrichment of promoters in histone marks [13], we observe H3K4me3 enrichment on hypomethylated promoters, either in a monovalent state (55% of the hypomethylated promoters) or in combination with H3K27me3 (Figure 5b). An implication of these findings is that a hypomethylated promoter marked by H3K4me3, without or with H3K27me3, is in a transcriptionally permissive state, even though the gene is not necessarily transcribed.

### Relationship between pre-MBT promoter marking by DNA methylation and modified histones, and post-ZGA gene expression

**Figure 6:**
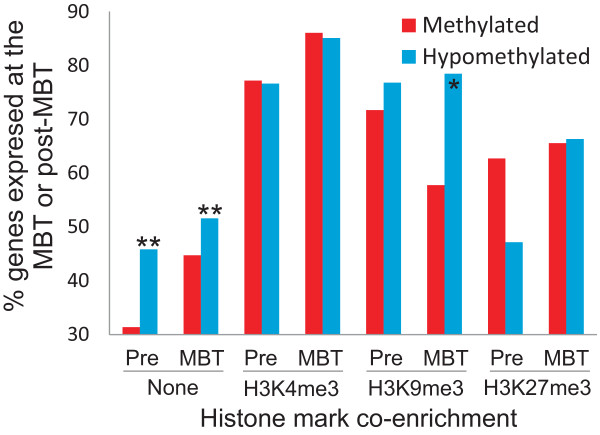
**Promoter marking by modified histones before ZGA onset is associated with enhanced gene expression potential after ZGA**. Graph shows the percentage of expressed genes (≥5 RNA-seq reads) at or after the MBT (post-ZGA) as a function of promoter DNA methylation state and marking by H3K4me3, H4K9me3 or H3K27me3 at the pre-MBT or MBT stages. **P < 10^-5^; *P < 0.001; ^-^*P *= 0.058 (Chi-square test with Yate's correction).

We next determined whether promoter DNA methylation or hypomethylation at the pre-MBT and MBT stages was preferentially associated with post-ZGA gene expression or absence thereof. First, although both methylated and hypomethylated genes can be expressed after ZGA, a higher proportion of genes hypomethylated pre-MBT or at the MBT are expressed after ZGA, compared to methylated genes (Figure 6; *P *< 10^-4^). Thus, pre-MBT promoter hypomethylation correlates with enhanced expression potential after ZGA. Second, pre-MBT enrichment in H3K4me3, H3K9me3 or H3K27me3 is associated with a higher proportion of expressed genes post-ZGA than of genes lacking these histone marks (Figure 6; *P *< 10^-4^); this is regardless of the histone mark and of promoter methylation state. Thus, histone marking pre-MBT may confer a propensity for post-ZGA gene expression. However, this propensity is affected by the nature of the histone modification, with H3K9me3 on methylated promoters at pre-MBT and MBT stages being associated with a reduced proportion of expressed genes post-ZGA (Figure 6; *P *< 0.0001). Thus, promoter hypomethylation pre-MBT confers a greater propensity for gene activation after ZGA than the methylated state.

### Relationship between sperm, embryo and fibroblast promoter methylation

**Figure 7:**
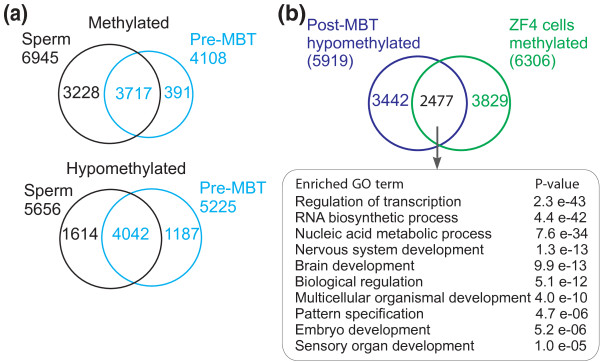
**Relationship between promoter methylation in sperm and embryos**. **(a) **Venn diagram analyses of methylated and hypomethylated genes in sperm and in pre-MBT embryos. **(b) **Venn diagram analysis of genes hypomethylated in post-MBT embryos and methylated in ZF4 cells. Top 10 enriched GO terms for these genes are shown.

Zebrafish sperm is hypermethylated relative to the oocyte [7] and has a methylation profile similar to differentiated cells in most of the genome [27]. The dynamics of sperm promoter methylation after fertilization, however, is unknown. To address this issue, we determined zebrafish sperm methylation patterns by MeDIP-ChIP and compared sperm and embryo DNA methylation profiles by intersecting genes methylated in sperm and in pre-MBT embryos. We detected 6,945 methylated and 5,856 hypomethylated genes in zebrafish sperm (Figure 7a; Additional file 1) implicated in signal transduction and developmental functions, respectively (Additional file 2). Both methylated and hypomethylated regions in sperm encompass entire gene clusters or stand-alone genes, as observed previously using a similar approach [27]. Interestingly, we note that substantial demethylation occurs between fertilization and the pre-MBT stage examined, as judged by >40% of promoters methylated in sperm being unmethylated in embryos (Figure 7a); these promoters may conceivably escape post-fertilization remethylation, at least at the stages examined. Nonetheless, 90% of genes methylated in pre-MBT embryos are methylated in sperm (Figure 7a). This suggests that these promoters are demethylated after fertilization, and remethylated by the 256-cell stage; alternatively, for these genes, the methylated state of sperm is retained after fertilization. In addition, we note a more robust preservation of the hypomethylated state between sperm and embryos compared to the methylated state (Figure 7a), consistent with the idea of a pre-patterning of early developmental gene expression already in sperm [27].

Although the hypomethylated state is largely conserved during early development, this may, however, not be the case upon differentiation. A comparison of methylation levels for all genes between embryos and cultured ZF4 fibroblasts using two-dimensional scatter plots reveals methylation of genes not methylated in post-MBT embryos (Figure 1b). In fact, we find that 42% of hypomethylated genes in post-MBT embryos are methylated in ZF4 fibroblasts (Figure 7b). Notwithstanding the fact that ZF4 cells are cultured *in vitro*, in contrast to embryos, these data are consistent with a differentiation-induced methylation. Interestingly, genes methylated in fibroblasts encode developmentally regulated transcription factors involved in pattern specification and nervous system development (Figure 7b; Additional file 2), functions that are repressed in fibroblasts. These genes notably include members of the homeobox genes *dlx*, *fox*, *grx*, *hox*, *irx*, *lhx*, *nkx*, *pax*, *pdx *and *tbx *(Additional files 5 and 6). In contrast, CG-rich housekeeping genes are not methylated in ZF4 cells (for example, *bact1*; Additional file 6). Our data are therefore consistent with a loss of sperm hypomethylation of developmentally regulated promoters in somatic tissues, as recently shown in a MeDIP-chip comparative analysis of zebrafish sperm and muscle [27]. Interestingly, GO terms for these genes strongly overlap with GO terms associated with genes found within clusters of three or more CGIs (Additional file 5), which encode developmentally regulated transcription factors [28]. Thus, DNA methylation elicited by differentiation may favor CGI clusters (including developmental regulators; for example, *hox *genes) over genes containing single CGIs, which primarily harbor housekeeping functions (for example, *bact1*). The hypomethylated state of CGI clusters in sperm and embryos may therefore constitute an epigenetic pre-patterning of a subsequent differentiation program.

## Discussion

We report here a DNA methylation profiling of promoters and 5' end of genes in sperm and embryos developing through the MBT, a period of intense chromatin remodeling [7,13,14]. Both sperm and embryonic genomes are marked by areas of methylation and hypomethylation; yet a striking observation is the significant proportion of genes methylated in sperm and unmethylated in pre-MBT, MBT and post-MBT embryos. In the context of the global demethylation and remethylation of the zebrafish genome that follows fertilization [7], it is possible that these genes escape post-fertilization remethylation. Their hypomethylated state argues for transcriptional permissiveness, in line with their homeostatic and developmental functions.

Nevertheless, nearly all genes methylated in embryos are also methylated in sperm. This overlap is consistent with a transgenerational inheritance of DNA methylation states through fertilization. This view is supported by the incomplete erasure of parental promoter methylation in the mouse [2,3] and the lack of global demethylation in *Xenopus *embryos [29]. Our data are also compatible with a resetting of DNA methylation after fertilization, as promoter methylation changes reported here parallel global methylation changes [7], implying demethylation and remethylation phases. This interpretation is in line with post-fertilization methylation events observed in mouse [2,3] and *Xenopus *[6] embryos. A demethylation/remethylation model would nevertheless argue that a site-specific methylation determinant be perpetuated through fertilization. A high CG content may be a key genetic determinant for inheritance of the hypomethylated state, while determinants of the methylation state may speculatively include non-coding RNAs, which have been identified in sperm [30,31]. This exciting question remains to be examined closely.

A large fraction of genes hypomethylated in embryos are also hypomethylated in sperm ([27] and this paper). Because these genes are enriched in transcription regulation and developmental functions, hypomethylation may confer, already in sperm, an instructive role for the developmental gene expression program. Similarly, hypomethylation and homeostatic functions of maternally expressed genes [10] support a model of an instructive role of the hypomethylated state in female gametes. One such key instructive function may be the targeting of H3K4me3 after fertilization, which takes place on DNA hypomethylated regions, preferentially occurs on maternally expressed genes, and has been proposed to confer a propensity for transcriptional activation after ZGA onset [13].

Several loci of developmentally important genes stand out as hypomethylated in sperm and embryos, but are methylated in ZF4 fibroblasts. These genes are largely CG-rich. Interestingly, genes associated with clusters of three or more CGIs have been shown to be genetically marked in a manner that distinguishes them from genes associated with a single CGI [28]. Genes in CGI clusters mostly encode transcription factors with sequence-specific DNA binding properties (such as *hox *genes), while single CGI genes mainly harbor housekeeping functions [28]. We find that genes in CGI clusters overlap with regions hypomethylated only in embryos and are methylated in fibroblasts; this is strongly suggestive of a net methylation gain on developmentally regulated genes that no longer need to be expressed in fibroblasts, in order to elicit long-term repression of the developmental functions these genes are involved in. In contrast, genes with single CGIs hypomethylated in embryos remain hypomethylated in fibroblasts, consistent with the cellular homeostatic functions of these genes (these observations also incidentally indicate that methylation detected in ZF4 fibroblasts is not a mere consequence of their cultured state, but reflects a differentiation process). Differential methylation of genes associated with single versus multiple CGIs elicited by differentiation may be due to developmental stage-dependent or tissue-specific alternative Dnmt usage [16,17]. An intriguing question is whether genetic determinants also exist for other types of epigenetic marks and if such a genomic component might have a stronger impact on the epigenome during early development than in differentiated cells.

We previously showed that a greater proportion of maternal genes than zygotic genes are marked by H3K4me3 after ZGA [13]. Our DNA methylation profiling provides an epigenetic basis for this observation by showing that maternal promoters are enriched in hypomethylated HCPs, and that most hypomethylated promoters are marked by H3K4me3. Indeed, in mammalian cells CpG density within CGIs correlates with H3K4me3 levels [32], and unmethylated CGIs are sufficient to target SETD1-mediated H3K4me3 in concordance with recruitment of the adaptor protein CFP1 [26]. Our RNA-seq data indicate that, in zebrafish, both homologues of the human H3K4 histone methyltransferase *SETD1A *and *SETD1B *genes are maternally expressed and transcripts remain detected at high levels until the MBT, after which they are degraded (data not shown) [10]. Interestingly, a similar transcript profile occurs for *cfp1*, suggesting that the Setd1/Cfp1 histone methyltransferase complex may operate post-fertilization in zebrafish, for H3K4 trimethylation on hypomethylated promoters in the pre-MBT period.

Cohorts of genes differentially expressed through the MBT display distinct methylation patterns related to CG content rather than transcription status. Maternal genes are enriched in HCPs, suggesting that a sequence determinant may define their maternal status. Their hypomethylated state may enable transcription during oogenesis; however, we show that their transcriptional fate is not determined by methylation state. In addition, zygotic genes contain a higher proportion of methylated promoters than maternal genes, nearly as high as non-expressed genes, consistent with a lack of correlation between promoter methylation and embryonic gene expression (see also [6]). In the mouse, however, promoter methylation seems to restrict gene activation, until demethylation associated with tissue-specific differentiation [2]. Anamniote vertebrate development may thus mainly rely on changes in histone modifications rather than DNA methylation to regulate gene expression changes at the onset of ZGA [14,33,34]. Additional experiments will be required to dissect the genetic and epigenetic determinants of chromatin states and gene expression patterns during zebrafish development.

## Conclusions

Our results suggest an epigenetic pre-patterning of early zebrafish developmental gene expression potential by a state of hypomethylation and marking by H3K4me3 of CG-rich promoters of developmental importance. Our data are also consistent with the view of a transgenerational inheritance of DNA methylation states from gametes to embryos.

## Materials and methods

### MeDiP

The MeDIP-chip protocol of Schübeler and colleagues [23] and slightly modified by us [35] was optimized for early stage zebrafish embryos with a high yolk/DNA ratio. Embryos at pre-MBT, MBT and post-MBT stages were collected at 2.5, 3.5 and 5.3 hpf, respectively. Chorions were removed with 1 mg/ml pronase in phosphate-buffered saline for 5 to 10 minutes at 37°C and embryos were washed three times in egg water (60 μg/ml Instant Ocean*^® ^*sea salts). Yolk was removed in 1 ml ice-cold de-yolking buffer (55 mM NaCl, 1.25 mM NaHCO_3_, pH 7.2) and sedimentation at 300 g for 30 s. The supernatant was discarded and de-yolking repeated once. Embryos were flash-frozen in liquid N_2_ as dry pellets. Frozen embryos were partially thawed and lysed in 300 µl of cell lysis buffer (50 mM Tris-HCl, pH 8.0, 10 mM EDTA, 1% (w/v) SDS). Lysates were sonicated with a Bioruptor (Diagenode; Liège, Belgium) for 5 minutes (30 s on/off) at high power. Proteins were digested with 0.5 µg/ml proteinase K on a Thermomixer at 55°C for 1 h, followed by overnight digestion after a second addition of proteinase K. DNA was isolated by phenol-chloroform extraction and ethanol precipitation using 10 µl acrylamide carrier, and RNase-treated. DNA was re-sonicated to 300 to 1,000 bp fragments.

Duplicate MeDIPs were performed as described [35] using 4 µg DNA (at 8 ng/µl), 5 µl anti-5-methylcytosine antibody (10 ng/µl; Mab-006-100; Diagenode) and Dynabeads M-280 sheep anti-mouse IgG (Life Technologies; Carlsbad, CA, USA). For negative controls, we used 10 ng/µl mouse IgG. Samples were washed, deproteinized and DNA purified. Input DNA was fragmented and treated as above without immunoprecipitation. MeDIP and input DNA (150 ng each) were amplified (WGA-2; Sigma-Aldrich; St Louis, MO, USA), cleaned up, eluted and processed for array hybridization.

### Array hybridization and data analysis

Input and MeDIP DNA fragments labeled with Cy3 and Cy5, respectively, were hybridized to a Roche-Nimblegen 2.1-million probe array described earlier [18]. Signal intensities were centered on zero using NimbleScan [36]. From scaled log_2_ MeDIP/input ratios, a 750-bp window was placed around each consecutive probe and a one-sided KS test was applied. Resulting score for each probe was the *P*-value from the windowed test around that probe. Using NimbleScan, methylated peak data were generated from *P*-values by searching for at least two probes with a *P*-value cut-off of ≤0.01. A promoter region was scored as hypermethylated if it overlapped with at least one peak, in both replicates. Data were viewed using Nimblegen SignalMap. Correlation of log_2_ MeDIP/input DNA ratios between replicates and between stages were computed using MaxSixty values as described earlier [18]. Metagenes of average methylation enrichment over the tiled region were calculated [18] using genes with high enrichment probability (KS ≤0.01). GO term enrichment was calculated using Bioconductor GOstats. For consistency with our previous analysis of histone modification on zebrafish promoter regions using similar arrays [13], MeDIP analysis was restricted to the -5 to +1 kb region relative to the TSS, unless otherwise stated.

### Detection of hypomethylated promoters

The methylation peak detection algorithm used in this study relies on the assignment of a *P*-value to each probe ratio using a KS test; low *P*-values translate to a high probability that the probe is enriched. Similarly, a high *P*-value is expected to indicate that the probe is likely normal (non-enriched). We therefore scored a promoter as hypomethylated if all probes in a -1 to 0 kb window upstream of the TSS had KS scores indicating a chance of ≥0.99 of being non-enriched in both MeDIP replicates.

### Identification of HCPs and LCPs

To identify HCPs, we adapted the algorithm of Takai and Jones [20] as suggested earlier for zebrafish [21]. Using transcripts corresponding to the annotated NCBI gene IDs on the Nimblegen array, we determined the o/e CG ratios in the entire 1-kb upstream region. This 1-kb sequence was scanned using a 500-bp sliding window to identify a subsequence with an o/e CG ratio ≥0.65 and a CG content >0.30. This approach combines the relatively stringent method of Takai and Jones [20] while taking into account the mean CG content of 0.3 identified in promoters (Additional file 3). Promoters containing a matching subsequence were scored as HCPs and those not matching any of these criteria were scored as LCPs. CG content analysis was done using accessions from which corresponding RefSeq gene lists were derived. Graphical representation of CG density was done using CG plotter. Frequencies were calculated using a 500-bp window moving in 10-bp increments.

### Bisulfite sequencing

One microgram DNA sonicated as above was bisulfite converted using MethylEasy™ (Human Genetic Signatures; Sydney, Australia). Converted DNA was amplified by PCR using primers designed with Methprimer and positioned relative to the TSS (Additional file 7). PCR conditions were 95°C for 7 minutes and 35 cycles of 95°C for 1 minute, 52 to 55°C for 2 minutes and 72°C for 2 minutes, followed by 10 minutes at 72°C. PCR products were cloned into *E. coli *by TOPO TA cloning and sequenced. Sequenced DNA was quality-controlled using BiQ Analyzer [37].

### MeDIP-qPCR

DNA from duplicate MeDIPs and inputs was diluted to 7 ng/µl in H_2_O and qPCR performed using IQ SYBR^® ^Green [18] from 3 µl templates using indicated primers (Additional file 8). Data were analyzed relative to a standard curve and expressed as fold enrichment relative to input.

### ChIP-chip data

To apply histone modification profiles on DNA methylation patterns, we used our published data [13] available at the NCBI Gene Expression Omnibus [GEO: GSE27314].

### Identification of developmental gene cohorts

RNA-seq data [GEO:GSE22830] were from a parallel study investigating the transcriptome of early zebrafish embryos [10]. For consistency with array data, reads were mapped to Zv7 using Bioscope v1.1 (Applied Biosystems; Carlsbad, CA, USA) and mapped reads counted for each RefSeq annotation. Expression data were mapped to NCBI gene IDs covered by the Nimblegen array for gene-level correlation of expression and MeDIP data. Developmental gene cohorts were defined as described [10]. Moreover, transcripts were considered as 'detected' if ≥5 length-adjusted RNA-seq reads were mapped to them, and as 'undetected' otherwise [13].

### Data access

MeDIP-chip data are available under [GEO:GSE33236]. Bisulfite sequencing data are publicly available on our URL [38].

## Abbreviations

CGI: CpG island; ChIP: chromatin immunoprecipitation; GO: gene ontology; H3: histone 3; HCP: high CpG promoter; hpf: hours post-fertilization; KS: Kolmogorov-Smirnov; LCP: low CpG promoter; MBT: mid-blastula transition; MeDIP: methylated DNA immunoprecipitation; o/e: observed/expected; qPCR: quantitative polymerase chain reaction; RNA-seq: RNA-sequencing; TSS: transcription start site; ZGA: zygotic gene activation.

## Competing interests

The authors declare that they have no competing interests.

## Authors' contributions

ISA designed the study, performed experiments, made figures and wrote parts of the manuscript. AHR designed and performed bioinformatics analyses, made figures and wrote parts of the methods. HA contributed to gene expression analysis. PA coordinated work with embryos and co-designed the study. PC designed the study, wrote the manuscript, made figures and supervised the work. All authors read and approved the final manuscript.

## Supplementary Material

Additional file 1 **Promoter DNA methylation in zebrafish embryos and sperm**. A figure showing aspects of promoter methylation in zebrafish embryos and sperm. Click here for file

Additional file 2 **Enriched GO terms for methylated and hypomethylated genes**. An Excel sheet of enriched GO terms for methylated and hypomethylated genes. Click here for file

Additional file 3 **CG content analysis of zebrafish promoters**. A figure showing the CG content analysis of zebrafish promoters. Click here for file

Additional file 4 **Partitioning of tiled regions reveals developmentally linked dynamic methylation upstream of TSS**. A figure showing methylation profiles in -1 to -1 kb regions around the TSS. Click here for file

Additional file 5 **Enriched GO terms of hypomethylated genes found in CGI clusters and methylated in ZF4 fibroblasts**. A table of enriched GO terms for hypomethylated genes in CGI clusters and that are methylated in ZF4 cells. Click here for file

Additional file 6 **Differential methylation of multiple versus single CGI promoters in embryos and ZF4 cells**. A figure showing methylation profiles of the *hoxa *and *bact1 *loci in post-MBT embryos and in ZF4 cells. Click here for file

Additional file 7 **Bisulfite sequencing primers used in this study**. A table of bisulfite sequencing primers used in this study. Click here for file

Additional file 8 **Primers used for MeDIP-qPCR validation**. A table of primers used for MeDIP-qPCR validation in this study. Click here for file
