# Supplementary material for: Developmental features of DNA methylation during activation of the embryonic zebrafish genome
Source: Genome Biol. 2012 Jul 25;13(7):R65. doi: 10.1186/gb-2012-13-7-r65 (PMC3491385; doi:10.1186/gb-2012-13-7-r65)
Supplement: Additional file 1 — Promoter DNA methylation in zebrafish embryos and sperm. A figure showing aspects of promoter methylation in zebrafish embryos and sperm. [file gb-2012-13-7-r65-S1.PDF]

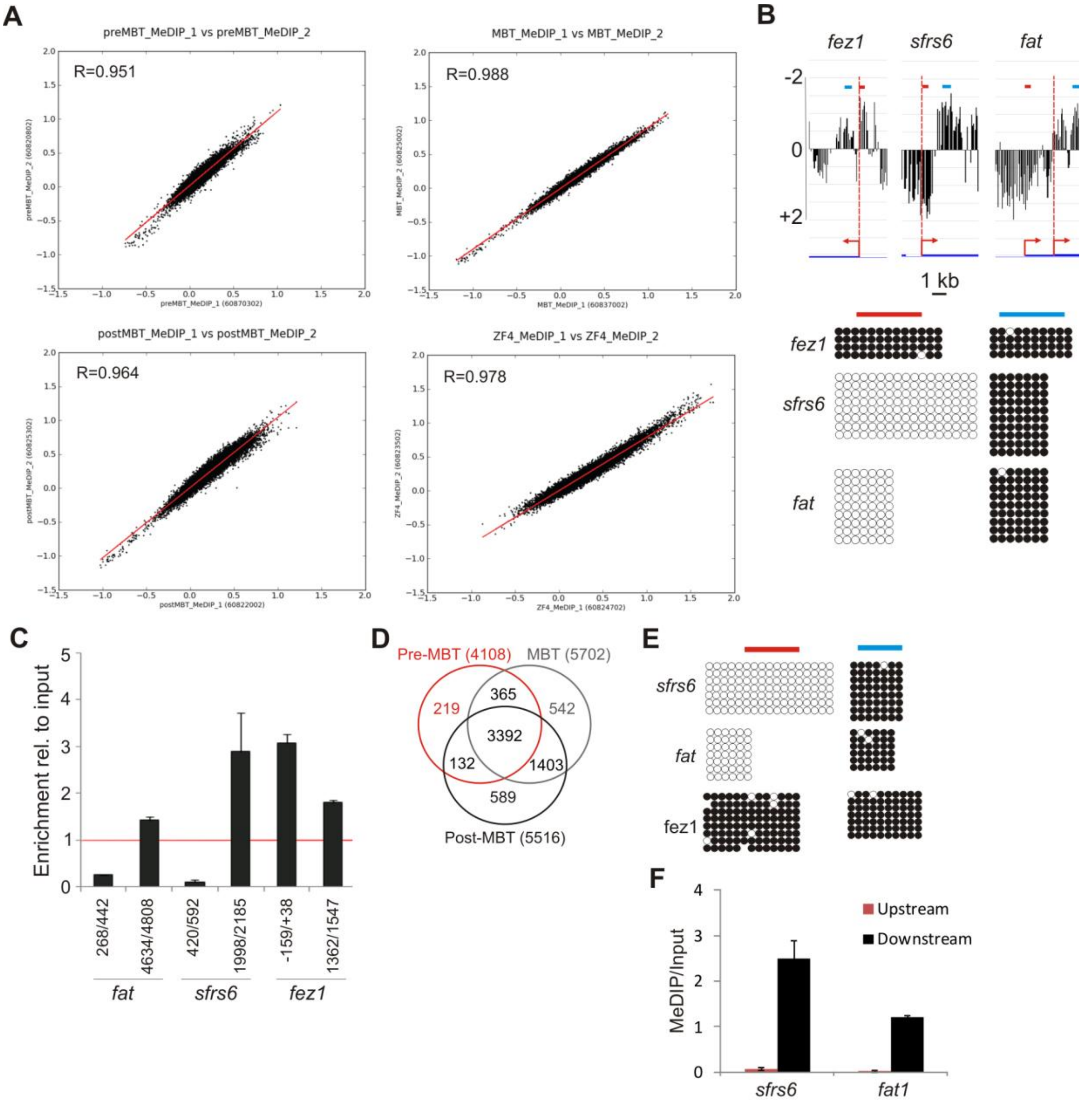

Additional file 1. Promoter DNA methylation in zebrafish embryos and sperm. (**A**) Replication analysis of MeDIP-chip in embryos. Two-dimensional scatter plots of MaxSixty values for MeDIP/input log_2 intensities in each of two independent MeDIP-chip replicates from pre-MBT, MBT and post-MBT embryos, and from ZF4 fibroblasts. Correlation coefficient (R) and regression line are shown. (**B**) MeDIP-chip profiles (top panels; log_2 MeDIP/input ratios) and bisulfite sequencing analysis (lower panels) of DNA methylation in indicated regions (red and blue bars) of the *fez1*, *sfrs6* and *fat* genes. (**C**) MeDIP-qPCR validation of MeDIP-chip data for indicated genes (mean ± s.e.m. from duplicate MeDIPs and duplicate qPCRs for each MeDIP). (**D**) Venn diagram of DNA methylation through the MBT period. Numbers of genes with a methylated promoter (-5 to +1 kb relative to TSS) are given. (**E, F**) Zebrafish sperm DNA methylation, analyzed by (**E**) bisulfite sequencing and (**F**) MeDIP-qPCR (mean±s.e.m. from duplicate MeDIPs). ● methylated cytosine; ○ unmethylated cytosine.

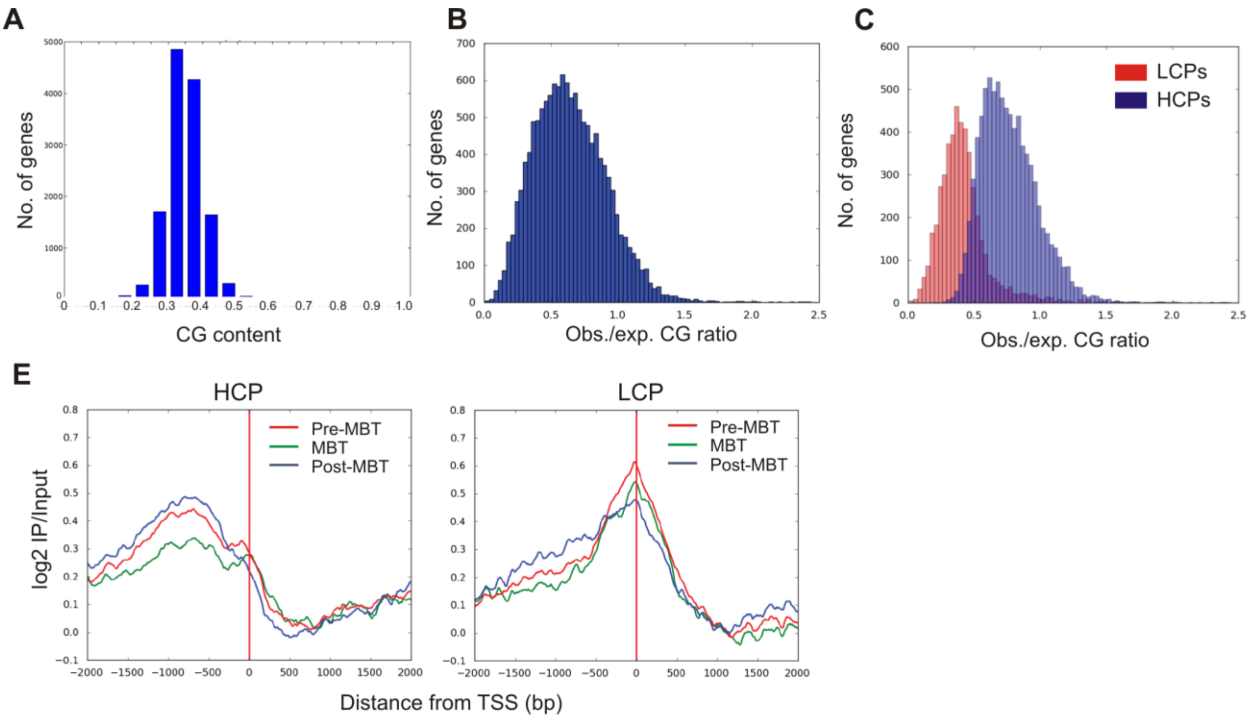

Additional file 3. CG content analysis of zebrafish promoters. (**A**) Distribution of the number of genes as a function of C+G content in the -1 to 0 kb region upstream of the TSS. (**B**) Observed/expected CG ratio for all zebrafish promoters. Numbers are those of NCBI gene IDs. (**C**) CpG content classification of promoters. HCPs, n=7914; LCPs, n=4341. Overlap includes promoters with a given o/e CG ratio but differing in C+G content and thereby partitioning into the LCP or HCP class. (**D**) Average methylation profiles of HCPs and LCPs at pre-MBT, MBT and post-MBT stages, over a -2 to +2 kb window on either side of the TSS.

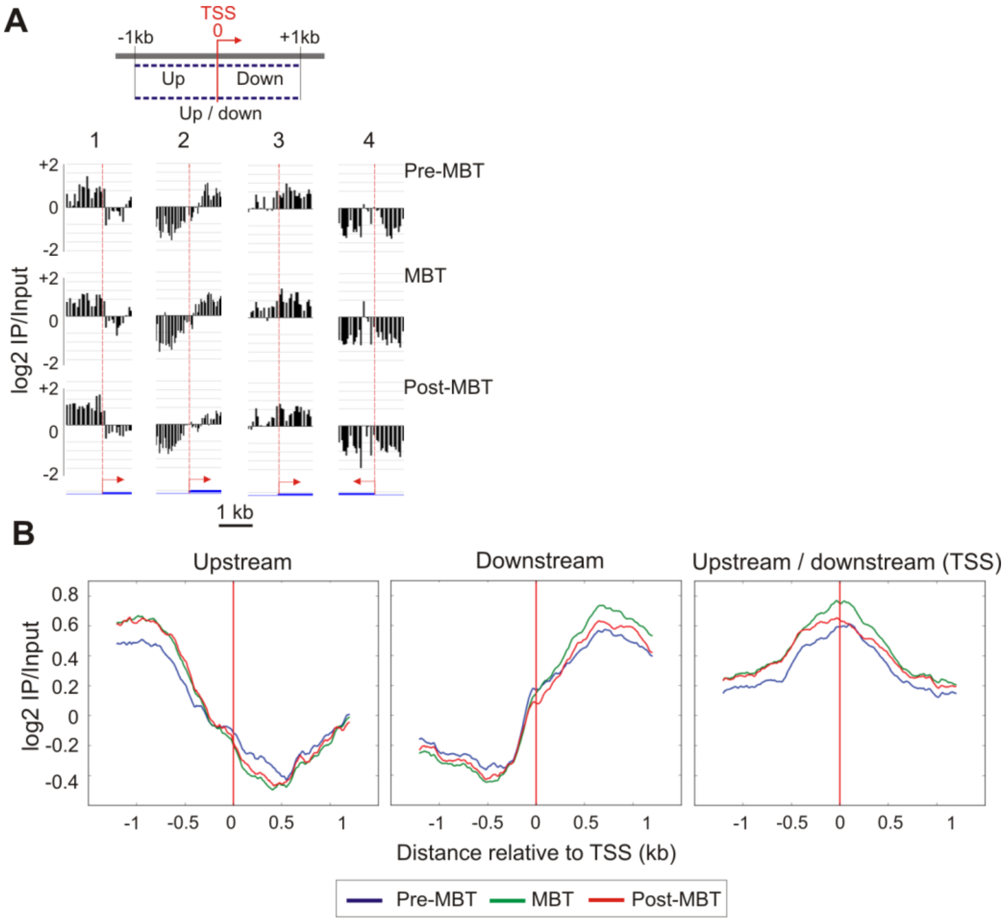

Additional file 4. Partitioning of tiled regions reveals developmentally-linked dynamic methylation upstream of TSS. (**A**) Partitioning of the -1 to +1 kb region relative to the TSS, and browser representation of (1) upstream methylation (ENSDART00000037383), (2) downstream methylation (*zgc:56306*), (3) up/downstream methylation (*atf7ip*) and (4) no methylation (*lef1*). Blue marls (bottom track) indicates genes; red arrows indicate TSSs. (**B**) Metagene profiles of average DNA methylation in upstream, downstream and upstream/downstream partitions at the pre-MBT, MBT and MBT stages.

Additional file 5. Enriched GO terms of embryo-hypomethylated genes found in CGI clusters, and methylated in ZF4 fibroblasts

**GO terms***	
GO:0006139	nucleobase, nucleoside, nucleotide and nucleic acid metabolic process
GO:0048699	generation of neurons
GO:0043170	macromolecule metabolic process
GO:0032774	RNA biosynthetic process
GO:0007399	nervous system development
GO:0007389	pattern specification process
GO:0006355	regulation of transcription, DNA-dependent
GO:0010468	regulation of gene expression
GO:0009790	embryonic development
GO:0010467	gene expression
GO:0045449	regulation of transcription
GO:0048731	system development
GO:0050794	regulation of cellular process
GO:0050789	regulation of biological process
GO:0007420	brain development

*GO terms enriched in CGI clustered genes.

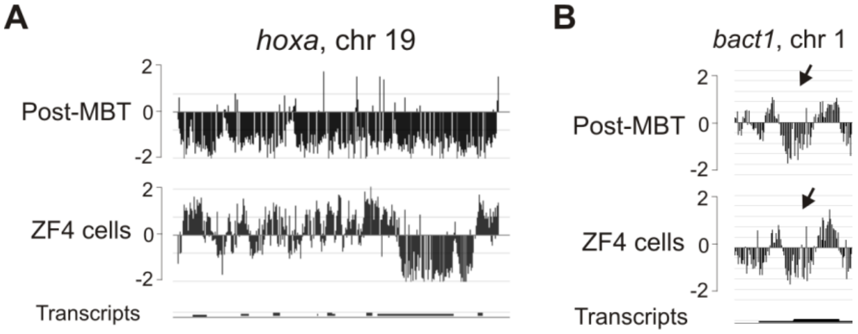

Additional file 6. Differential methylation of multiple vs. single CGI promoters in embryos and ZF4 cells. (**A**) Hypomethylated domain over the *hoxa* locus in embryos but not in ZF4 cells (region 10,475,000-10,552,000 on chromosome 19). (**B**) Maintenance of hypomethylation (arrow) of the *bact1* promoter in embryos and ZF4 cells (region 4,467,000-4,476,000 on chromosome 1).

Additional file 7. Bisulfite sequencing primers used in this study

Gene	Forward primer (F) 5'→3'<br>Reverse primer (R) 5'→3'	Position* rel. to TSS (nt)	Annealing temp. (°C)
fat	F1: GTGTAAATTGGTTAGTTAATTGTTTTAAAT	+196	55
	R1: TACTATCAAAAAAACCTCTCTTTAAC	+421	
	F2: TATTTGTTGGGGTTAGGATTGTTTA	+4492	55
	R2: ACTCTCACTAATTTCCACAAAATAC	+4806	
sfrs6	F1: GGGAGAAGGATATTTAGAGGTTTTT	+131	55
	R1: CCAATTCAAAAATCTTACTACTTTCAAA	+481	
	F2: AAATGGGGATTTTTTTTAAAATTGT	+2075	55
	R2: CAATCATCTTAACCTCAATAAACTC	+2441	
fez1	F1: ATGAATATGTAGGGATTTGGTTTTT	-332	55
	R1: ATAAATCCACCATCAATCAAAATAAA	-39	
	F2: TTGGGAATTAGTTATGTAGATAAAATAATA	+1180	52
	R2: TAATCAACCTTACAACAAAAAAAATC	+1546	
bact1	F1: AATTTAGAATTTGAATTTTAAGTAATTAGT	-68	52
	R1: TAAAATAAATCACAACTTTATAACC	+76	
pou5f1	F1: TGTTTTTTTTATTTTTTAAATATTTTTG	-718	55
	R1: TACTTTCACTTACATTTTTACTATTCTTTT	-535	
klf4	F1: AGATAAATTGATTTTTTTTGTAATTTATAT	-279	55
	R1: ATTTAACTAAATATCTACCTATCCCATTAT	-1	

* As per Zv9 assembly.

Additional file 8. Primers used for MeDIP-qPCR validation

Gene	Forward primer (F) 5'→3'<br>Reverse primer (R) 5'→3'	Position* rel. to TSS (nt)	Annealing temp. (°C)
fat	F1: GATCGTAGCATGGCAGAG	+268	60
	R1: CAGTCCGTCAATTCCTATTCA	+442	
	F2: CTGGAGGTGATGCTGAGA	+4634	60
	R2: AACTGTGGACTGTTGTCATTAG	+4808	
sfrs6	F1: AAATGGCGGATTGTTTGTGT	+420	60
	R1: GTCACGTCAGCACCTTCA	+592	
	F2: TGCCTGTCTTCTGGATGGA	+1998	60
	R2: AGTTAGTCACTCTCCCTGATGTT	+2185	
fez1	F1: GGCTGGCTTAGACAAGAC	-159	60
	R1: CCTCCTATTCCGATATAGTCAGA	-38	
	F2: CATCCATACAACACTCATTCAC	+1362	60
	R2: TTAATCAGCCTTACAACAAGAAG	+1547	

* As per Zv9 assembly.
