# Supplementary material for: Developmental features of DNA methylation during activation of the embryonic zebrafish genome
Source: Genome Biol. 2012 Jul 25;13(7):R65. doi: 10.1186/gb-2012-13-7-r65 (PMC3491385; doi:10.1186/gb-2012-13-7-r65)
Supplement: Additional file 3 — CG content analysis of zebrafish promoters. A figure showing the CG content analysis of zebrafish promoters. [file gb-2012-13-7-r65-S3.PDF]

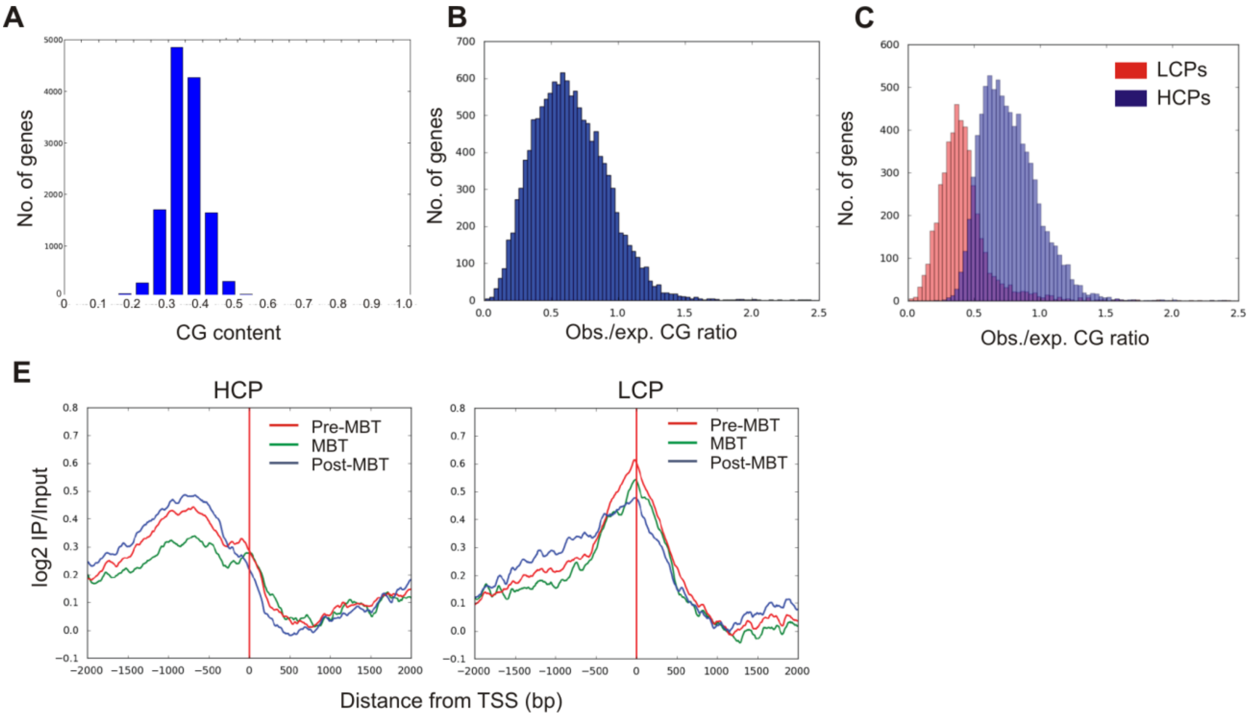

Additional file 3. CG content analysis of zebrafish promoters. (**A**) Distribution of the number of genes as a function of C+G content in the -1 to 0 kb region upstream of the TSS. (**B**) Observed/expected CG ratio for all zebrafish promoters. Numbers are those of NCBI gene IDs. (**C**) CpG content classification of promoters. HCPs, n=7914; LCPs, n=4341. Overlap includes promoters with a given o/e CG ratio but differing in C+G content and thereby partitioning into the LCP or HCP class. (**D**) Average methylation profiles of HCPs and LCPs at pre-MBT, MBT and post-MBT stages, over a -2 to +2 kb window on either side of the TSS.
