# Supplementary material for: Developmental features of DNA methylation during activation of the embryonic zebrafish genome
Source: Genome Biol. 2012 Jul 25;13(7):R65. doi: 10.1186/gb-2012-13-7-r65 (PMC3491385; doi:10.1186/gb-2012-13-7-r65)
Supplement: Additional file 4 — Partitioning of tiled regions reveals developmentally linked dynamic methylation upstream of TSS. A figure showing methylation profiles in -1 to -1 kb regions around the TSS. [file gb-2012-13-7-r65-S4.PDF]

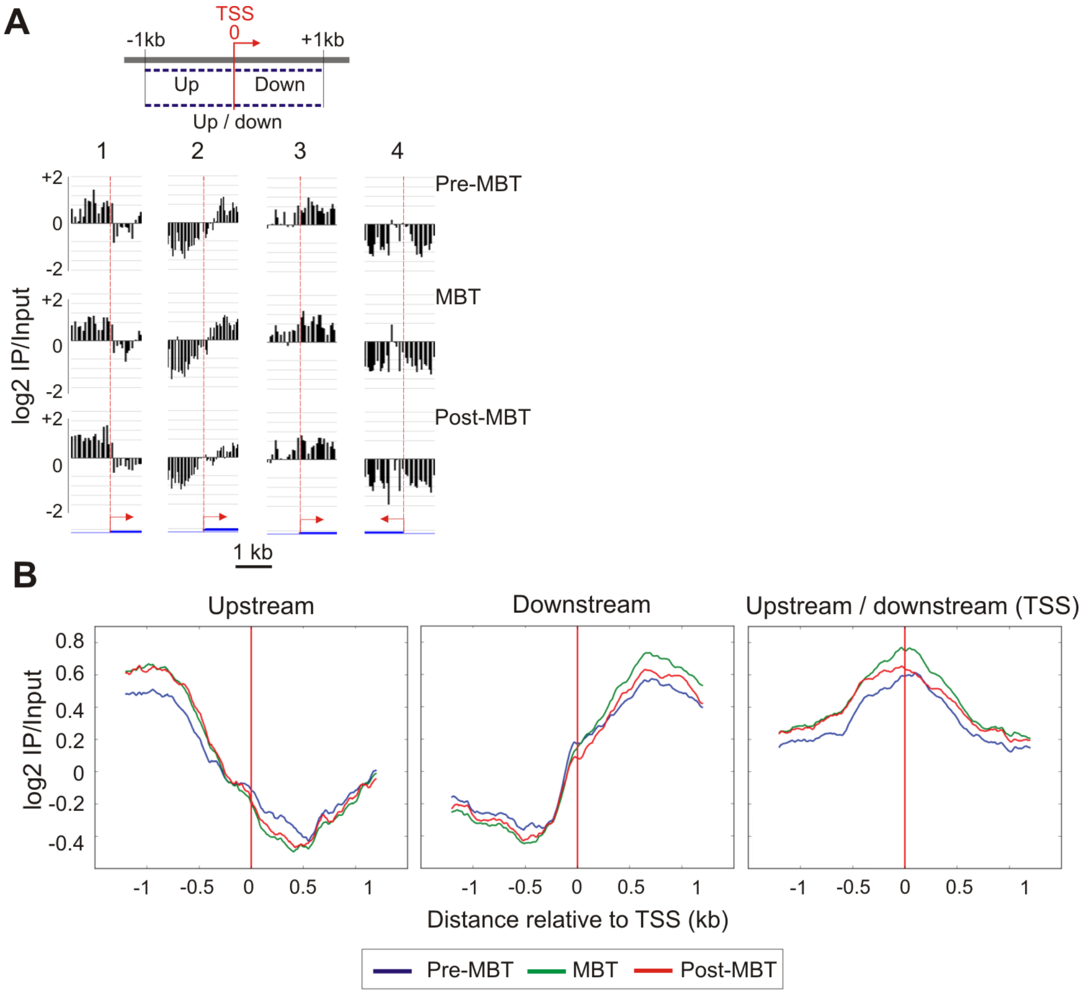

Additional file 4. Partitioning of tiled regions reveals developmentally-linked dynamic methylation upstream of TSS. (**A**) Partitioning of the -1 to +1 kb region relative to the TSS, and browser representation of (1) upstream methylation (ENSDART00000037383), (2) downstream methylation (*zgc:56306*), (3) up/downstream methylation (*atf7ip*) and (4) no methylation (*lef1*). Blue marls (bottom track) indicates genes; red arrows indicate TSSs. (**B**) Metagene profiles of average DNA methylation in upstream, downstream and upstream/downstream partitions at the pre-MBT, MBT and MBT stages.
