# Supplementary material for: Developmental features of DNA methylation during activation of the embryonic zebrafish genome
Source: Genome Biol. 2012 Jul 25;13(7):R65. doi: 10.1186/gb-2012-13-7-r65 (PMC3491385; doi:10.1186/gb-2012-13-7-r65)
Supplement: Additional file 5 — Enriched GO terms of hypomethylated genes found in CGI clusters and methylated in ZF4 fibroblasts. A table of enriched GO terms for hypomethylated genes in CGI clusters and that are methylated in ZF4 cells. [file gb-2012-13-7-r65-S5.PDF]

Additional file 5. Enriched GO terms of embryo-hypomethylated genes found in CGI clusters, and methylated in ZF4 fibroblasts

**GO terms***	
GO:0006139	nucleobase, nucleoside, nucleotide and nucleic acid metabolic process
GO:0048699	generation of neurons
GO:0043170	macromolecule metabolic process
GO:0032774	RNA biosynthetic process
GO:0007399	nervous system development
GO:0007389	pattern specification process
GO:0006355	regulation of transcription, DNA-dependent
GO:0010468	regulation of gene expression
GO:0009790	embryonic development
GO:0010467	gene expression
GO:0045449	regulation of transcription
GO:0048731	system development
GO:0050794	regulation of cellular process
GO:0050789	regulation of biological process
GO:0007420	brain development

*GO terms enriched in CGI clustered genes.
