# Supplementary material for: Developmental features of DNA methylation during activation of the embryonic zebrafish genome
Source: Genome Biol. 2012 Jul 25;13(7):R65. doi: 10.1186/gb-2012-13-7-r65 (PMC3491385; doi:10.1186/gb-2012-13-7-r65)
Supplement: Additional file 6 — Differential methylation of multiple versus single CGI promoters in embryos and ZF4 cells. A figure showing methylation profiles of the hoxa and bact1 loci in post-MBT embryos and in ZF4 cells. [file gb-2012-13-7-r65-S6.PDF]

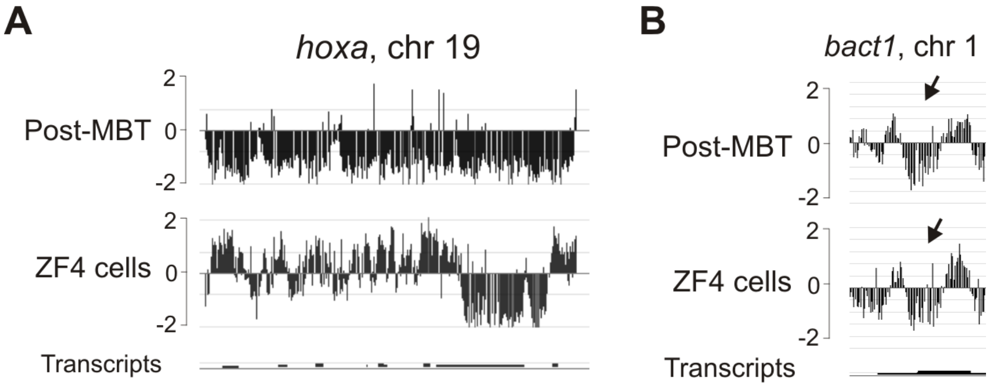

Additional file 6. Differential methylation of multiple vs. single CGI promoters in embryos and ZF4 cells. (**A**) Hypomethylated domain over the *hoxa* locus in embryos but not in ZF4 cells (region 10,475,000-10,552,000 on chromosome 19). (**B**) Maintenance of hypomethylation (arrow) of the *bact1* promoter in embryos and ZF4 cells (region 4,467,000-4,476,000 on chromosome 1).
