# Supplementary material for: Developmental features of DNA methylation during activation of the embryonic zebrafish genome
Source: Genome Biol. 2012 Jul 25;13(7):R65. doi: 10.1186/gb-2012-13-7-r65 (PMC3491385; doi:10.1186/gb-2012-13-7-r65)
Supplement: Additional file 7 — Bisulfite sequencing primers used in this study. A table of bisulfite sequencing primers used in this study. [file gb-2012-13-7-r65-S7.PDF]

Additional file 7. Bisulfite sequencing primers used in this study

Gene	Forward primer (F) 5'→3'<br>Reverse primer (R) 5'→3'	Position* rel. to TSS (nt)	Annealing temp. (°C)
fat	F1: GTGTAAATTGGTTAGTTAATTGTTTTAAAT	+196	55
	R1: TACTATCAAAAAAACCTCTCTTTAAC	+421	
	F2: TATTTGTTGGGGTTAGGATTGTTTA	+4492	55
	R2: ACTCTCACTAATTTCCACAAAATAC	+4806	
sfrs6	F1: GGGAGAAGGATATTTAGAGGTTTTT	+131	55
	R1: CCAATTCAAAAATCTTACTACTTTCAAA	+481	
	F2: AAATGGGGATTTTTTTTAAAATTGT	+2075	55
	R2: CAATCATCTTAACCTCAATAAACTC	+2441	
fez1	F1: ATGAATATGTAGGGATTTGGTTTTT	-332	55
	R1: ATAAATCCACCATCAATCAAAATAAA	-39	
	F2: TTGGGAATTAGTTATGTAGATAAAATAATA	+1180	52
	R2: TAATCAACCTTACAACAAAAAAATC	+1546	
bact1	F1: AATTTAGAATTTGAATTTTAAGTAATTAGT	-68	52
	R1: TAAAATAAATCACAACTTTATAACC	+76	
pou5f1	F1: TGTTTTTTTTATTTTTTAAATATTTTTG	-718	55
	R1: TACTTTCACTTACATTTTTACTATTCTTTT	-535	
klf4	F1: AGATAAATTGATTTTTTTTGTAATTTATAT	-279	55
	R1: ATTTAACTAAATATCTACCTATCCCATTAT	-1	

* As per Zv9 assembly.
