# Supplementary material for: Developmental features of DNA methylation during activation of the embryonic zebrafish genome
Source: Genome Biol. 2012 Jul 25;13(7):R65. doi: 10.1186/gb-2012-13-7-r65 (PMC3491385; doi:10.1186/gb-2012-13-7-r65)
Supplement: Additional file 8 — Primers used for MeDIP-qPCR validation. A table of primers used for MeDIP-qPCR validation in this study. [file gb-2012-13-7-r65-S8.PDF]

Additional file 8. Primers used for MeDIP-qPCR validation

Gene	Forward primer (F) 5'→3'<br>Reverse primer (R) 5'→3'	Position* rel. to TSS (nt)	Annealing temp. (°C)
fat	F1: GATCGTAGCATGGCAGAG	+268	60
	R1: CAGTCCGTCAATTCCTATTCA	+442	
	F2: CTGGAGGTGATGCTGAGA	+4634	60
	R2: AACTGTGGACTGTTGTCATTAG	+4808	
sfrs6	F1: AAATGGCGGATTGTTTGTGT	+420	60
	R1: GTCACGTCAGCACCTTCA	+592	
	F2: TGCCTGTCTTCTGGATGGA	+1998	60
	R2: AGTTAGTCACTCTCCCTGATGTT	+2185	
fez1	F1: GGCTGGCTTAGACAAGAC	-159	60
	R1: CCTCCTATTCCGATATAGTCAGA	-38	
	F2: CATCCATACAACACTCATTCAC	+1362	60
	R2: TTAATCAGCCTTACAACAAGAAG	+1547	

* As per Zv9 assembly.
